# Diplopia of Pediatric Orbital Blowout Fractures: A Retrospective Study of 83 Patients Classified by Age Groups

**DOI:** 10.1097/MD.0000000000000477

**Published:** 2015-01-30

**Authors:** Yun Su, Qin Shen, Ming Lin, Xianqun Fan

**Affiliations:** From the Department of Ophthalmology, Ninth People's Hospital, Shanghai Jiao Tong University, School of Medicine, Shanghai, China.

## Abstract

Orbital blowout fractures are relatively rare in patients under 18 years of age, but may lead to serious complications. We conducted this retrospective study to evaluate diplopia, clinical characteristics, and postoperative results in cases of orbital blowout fractures in the pediatric population.

Eighty-three patients, all less than 18 years old, with orbital blowout fractures, were divided into 3 groups by age: 0 to 6 years old, 7 to 12 years old, and 13 to 18 years old. The cause of injury, fracture locations, diplopia grades, ocular motility restrictions, enophthalmos, and postoperative results were reviewed from their records. Chi-square tests, Fisher's exact analyses, analyses of variance, and logistic regressions were performed to determine characteristics associated with diplopia, and to identify factors related to residual diplopia in pediatric patients.

The most common causes of injuries were traffic accidents in the 0 to 6 years old group, normal daily activities in the 7 to 12 years old group, and assaults in the 13 to 18 years old group. Floor fractures were the most common location in both the 0 to 6- and 7 to 12 years old groups, and medial-floor fractures were the most common location in the 13 to 18 years old group. The occurrence of preoperative diplopia was related to ocular motility restriction and enophthalmos, but not with the age group, the gender, the cause of injury, or the fracture locations. The time interval from injury to surgery was significant in the outcome of postoperative diplopia (*P* < 0.01). A statistical difference was also found in the recovery time from diplopia among the 3 age groups (*P* < 0.01).

The characteristics of orbital blowout fracture varied among the different age groups. It was related to 2 factors, the cause of injury and fracture locations, which probably resulted from structural growth changes and differences in daily habits. Children had a slower recovery from orbital fractures, and the younger the patient, the longer it took for recovery from diplopia after surgery.

## INTRODUCTION

Orbital fractures are relatively uncommon among children, accounting for 3% to 45% of all pediatric facial fractures.^1–4^ Injuries are not only limited to the orbit, but also extend to the adjacent facial skeletons, leading to aesthetic, functional, or even psychological deficits among the pediatric population.

Blowout fractures, defined as fractures of part of the orbital wall without any involvement of the orbital rim,^5,6^ are common in the pediatric age group, usually resulting in diplopia, disturbances of eye motility, and enophthalmos. Trapdoor fracture is a special type of fracture found especially in children, and can often include muscle and soft tissue injury. The cause of injury, clinical presentation, and management of orbital blowout fractures in adults have also been described.^7,8^ However, there is less data regarding orbital fractures in children. The unique anatomical and mechanical features of pediatric orbital fractures differentiate them from their adult counterparts. Even among individual children, clinical presentations may also be distinct at different ages, because of the development of orbital and maxillofacial anatomy. These features, together with concerns for future growth and development, require surgeons to differentially manage the pediatric population.

Previous studies emphasized either small case series, reviewing pediatric orbital fractures,^9,10^ or larger case series, focusing on facial fractures in general, where little information was collected about orbital injuries.^11,12^ In addition, there have been limited studies correlating growth features of different age groups, and fracture classification with pre- and postoperative diplopia in pediatric populations. The objective of this study was therefore to provide an overview of the demographics of this group of patients, and to describe patient characteristics in relation to the outcomes of diplopia at different age groups.

## MATERIALS AND METHODS

### Patients

This study was carried out according to the principles of the Declaration of Helsinki. Approvals from Shanghai Ninth People's Hospital Ethics Committee and informed consent were obtained. A retrospective review of 83 pediatric patients, with orbital blowout fractures, presenting to the Department of Ophthalmology, Shanghai Ninth People's Hospital, Affiliated Shanghai Jiao Tong University School of Medicine, between January 2008 and June 2013, was performed by 2 of the authors. All patients eligible for this study were less than 18 years of age, had suffered an orbital blowout fracture confirmed by computed tomography (CT), and had undergone surgical repair with follow-up for at least 12 months. Patients who had experienced previous surgical repair, visual loss or enophthalmos, corrected vision less than 0.3, complex orbital fractures, or bilateral orbital fractures were excluded. According to the standards of visual impairment raised by World Health Organization, corrected vision less than 6/18 (0.3) was defined as “low vision.”^13^ So we set 0.3 as a judging standard to reduce impact of low vision on the assessment of diplopia. Data regarding demographics, causes, time intervals between injury and operation, fracture walls, clinical presentations, management, and follow-ups were collected.

Our indications for surgery were as follows: clinically apparent extraocular muscle restriction with symptomatic diplopia that interfered with daily activities (within 30° of fixation), significant enophthalmos >2 mm at presentation, large fracture size (>50% of the orbital floor), positive forced duction testing, or other evidence of muscle or soft tissue entrapment (ie, CT scan).

Patients were subdivided into 3 age groups: 0 to 6, 7 to 12, and 13 to 18 years of age. Such classification was primarily based on maturity and growth of the facial and orbital skeleton, as well as additional characteristics in their social life. Facial skeletal growth and paranasal sinus pneumatization in children from 0 to 6 years of age involves a rapid growth phase.^14^ The cranial to facial ratio is the highest in this age group, and the group is more likely to be engaged in supervised activity. From 7 to 12 years of age, children are in an average growth phase, are starting school, and are beginning to interact more with others. Patients from 13 to 18 years of age undergo puberty, and become anatomically more similar to adults. As they are more independent, they confront more conflicts or at-risk activities.

### Preoperative Examinations

Forced duction testing was performed if patients were old enough to cooperate. Vision was measured with a visual testing chart if patients recognized letters, and for younger patients, a symbol chart was used as an approximate examination. Diplopia was examined by synoptophore if patients’ cooperation permitted, and a Hess screen test was also used for younger patients. We used conventional assessment approaches of diplopia in pediatric orbital fracture patients based on a previous study.^15^ The classifications of diplopia were as follows: Grade 0, no diplopia; Grade 1, diplopia only in peripheral vision (beyond 30°); Grade 2, diplopia in forward and/or downward direction (within 30°), without peripheral diplopia; and Grade 3, diplopia in all vision assessments.

### Surgical Techniques

All surgeries described in this study were performed by 1 surgical team, led by Dr. Xianqun Fan, who also performed the initial and follow-up evaluations. Surgical repairs were through a transconjunctival approach, with lateral canthotomy and cantholysis, if possible, to fully expose the orbital walls. Medpor^®^ (Porex Surgical, Fairburn, GA) was used in most patients. Hydroxyapatite (YHJ Science and Trade Co., Ltd., Beijing, China) and resorbable materials, RapidSorb^®^ (Synthes, West Chester, PA) were also used as implants.

### Statistical Analysis

Categorical variables were compared by chi-square tests. Fisher's exact test was used when exacted values were <5. Analysis of variance was applied in continuous variables, and the Fisher's least significant difference test was used between groups. We used binary logistic regression to identify factors for residual diplopia. A *P* value of <0.05 was considered significant. All statistical analyses were performed using SPSS 13.0 software (SPSS, Chicago, IL).

## RESULTS

### Demographics

**FIGURE 1:**
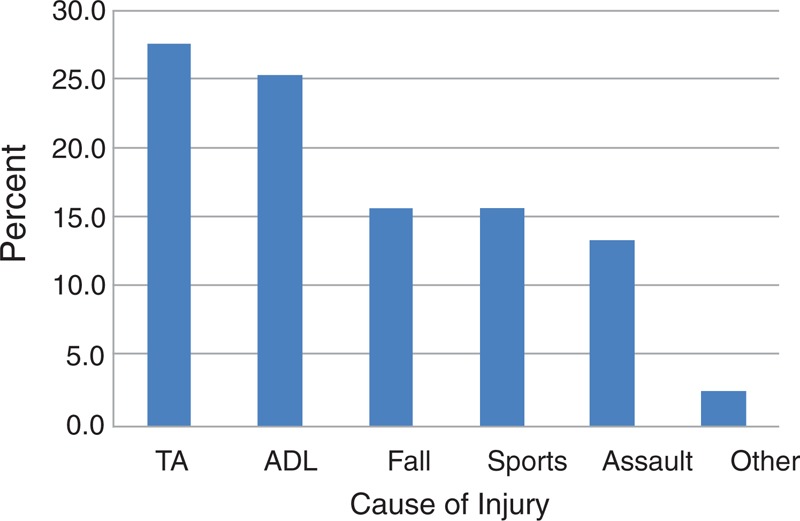
Causes of injury in all patients. Traffic accidents were the most common causes of injury, accounting for 27.7% of the injuries. The causes of 2 patients (2.4%) involved one unknown cause and the other declining to reveal. TA, traffic accident; ADL, activities of daily life.

**FIGURE 2:**
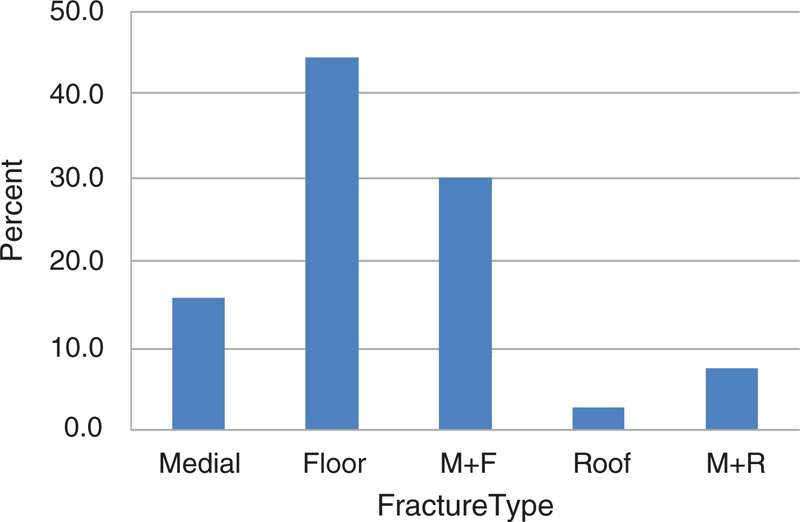
Percentage of all patients with a specific fracture. Orbital floor was the most common location of fractures (44.6%), and 2.4% of patients had orbital roof fractures. M + F, medial and floor; M + R, medial and roof.

Eighty-three patients, 59 male and 24 female, were included in this study. The mean age was 11.5 years (SD 4.7 years, range from 3 to 18 years of age). The causes of the orbital fractures are shown in Figure 1. Traffic accidents (including motor vehicle and bicycle accidents) were the most common causes of injury in pediatric patients (27/83, 27.7%). In the traffic accident group, 8 patients had bicycle accidents. Two of 83 (2.4%) patients had accidents from “other reasons,” including 1 unknown cause, and 1 declining to reveal. Figure 2 summarizes the specific orbital walls involved. The results showed that orbital floor fractures were by far the most common location, with 37/83 (44.6%) patients included in this group.

### Patient Characteristics by Age

#### 0 to 6 Years Old

**FIGURE 3:**
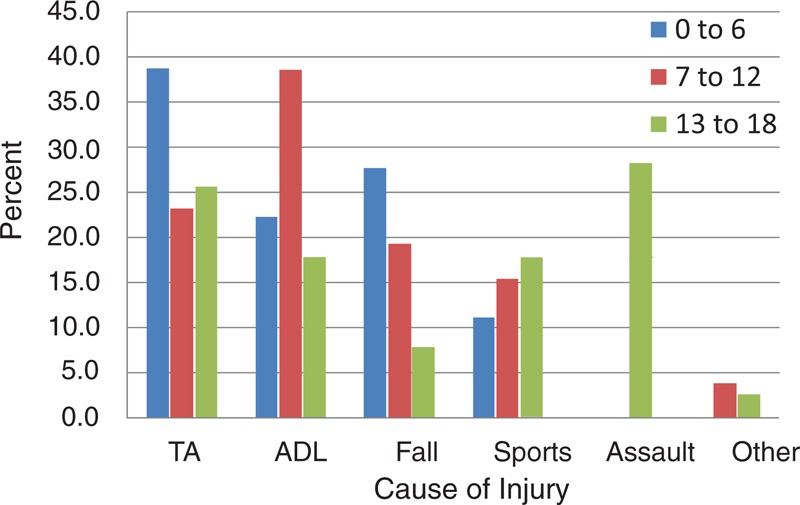
The cause of injury classified by age. Traffic accidents were the most common causes for the 0- to 6-year-old group. Activities of daily life were the most common causes in the 7- to 12-year-old group, and assault was the most common cause for the 13- to 18-year-old group. TA, traffic accident; ADL, activities of daily life.

**FIGURE 4:**
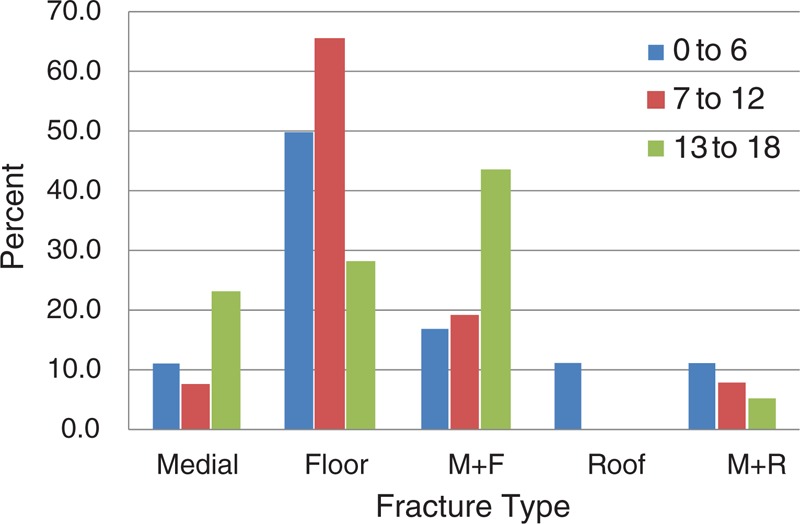
Percentage of fracture types classified by age. Orbital floor was the most common location of fractures for the 0- to 6-year-old group and 7- to 12-year-old group. Medial and floor fractures were the most common causes for the 13- to 18-year-old group. M + F, medial and floor; M + R, medial and roof.

This was the smallest of the 3 groups. Eighteen of 83 (21.7%) patients were involved in this group. The mean age was 4.6 years (SD 1.0, range from 3 to 6 years of age). Traffic accidents were the most common cause of injury (38.9%). There were no injuries related to assault. Orbital floor was the highest frequency (50.0%) among patients. Figures 3 and 4 show the causes of injury, and certain fracture types that were classified by age.

#### 7 to 12 Years Old

This group comprised 31.3% (26/83) of our population. The mean age was 9.1 years (SD 1.7, range from 7 to 12 years of age). The leading cause of injury was from activities in daily life (38.5%), unrelated to assault. The most common location was also the orbital floor in 65.4% of the patients.

#### 13 to 18 Years Old

This was the largest of the 3 groups (39/83, 47.0%). The mean age was 15.6 years (SD 1.6, range from 13 to 18 years of age). Assault was the most common cause of injury (28.2%). Medial-floor fractures accounted for nearly half (43.6%) of the cases in this age group.

### Clinical Assessment

Visual acuity at presentation was recorded for these patients. Seven patients were found to have impaired vision with visual acuity still no less than 0.3. Diplopia, ocular motility restriction, and enophthalmos were also noted at the initial examination. A large number of patients (71/83, 85.5%) had either Grade 2 or Grade 3 diplopia before surgery. All patients under 6 years of age had Grade 2 diplopia. The compositions of Grade 2 and Grade 3 diplopias were 88.5% and 76.9%, respectively. There seemed to be a decreasing trend in the preoperative diplopia grades in these 3 age groups. However, there were no statistically significant differences of preoperative diplopia between the age groups (*P* = 0.09). Table 1 shows the correlations between preoperative diplopia and clinical presentations. Eye motility restriction and enophthalmos were 2 parameters associated with diplopia before surgery. However, there was no statistical difference between preoperative diplopia and gender (*P* = 0.86), causes of injury (*P* = 0.06), or fracture locations (*P* = 0.63).

**TABLE 1:**
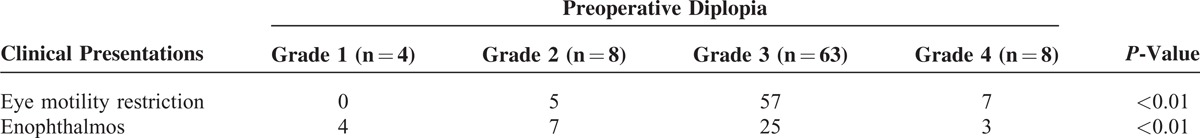
Association Between Preoperative Diplopia and Clinical Characteristics

The relationship of orbital wall fracture location to eye motility is shown in Table 2. Supraduction and infraduction limitation were the most common impairments in fractures involving the orbital floor.

**TABLE 2:**
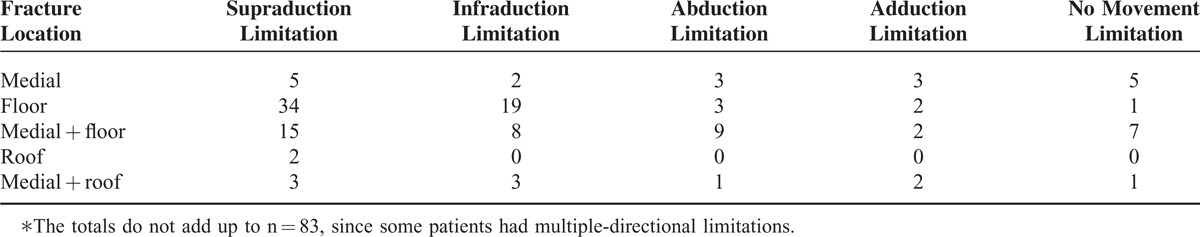
Relationship of Ocular Motility Limitations and Fracture Locations^∗^

Trapdoor fractures occurred in 15.7% (13/83) of children, with 7 patients less than 6 years of age, 5 patients between ages 7 to 12 years, and 1 patient over 13 years of age. The occurrence of trapdoor fracture was significantly correlated with the age group (*P* < 0.01). Table 3 illustrates the relationship of trapdoor fractures to preoperative diplopia. No relationship was found between trapdoor fractures and diplopia grades (*P* = 0.76).

**TABLE 3:**
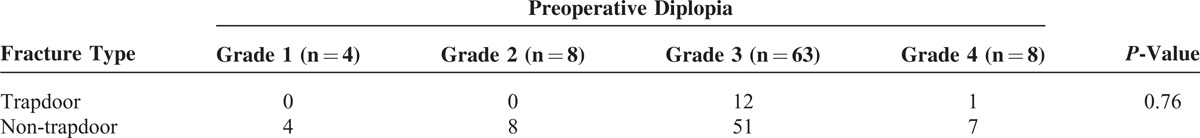
Relationship of Trapdoor Fractures to Preoperative Diplopia

The median interval time between injury and surgical intervention was 20 days, ranging from 7 days to 1 year. Sixty-six of 83 patients (79.5%) received surgical repair within 30 days. The interval time of 6 patients was as long as 1 year, because of poor general conditions after injury, such as spinal or visceral injuries.

The average follow-up time was 18.2 months (range, 12–72 months). Fortunately, no patients had postoperative complications, such as loss of vision, worsening of diplopia, and infection or extrusion of the orbital implant. Diplopia did not develop or worsen in any patient after surgery.

After surgical intervention, 66/79 (83.5%) of the patients who had preoperative diplopia completely recovered, 8 patients (10.1%) had remission of diplopia that involved only peripheral vision, and 5 patients still had obvious forwards or downwards diplopia. Table 4 lists the characteristics of these 5 patients.

**TABLE 4:**
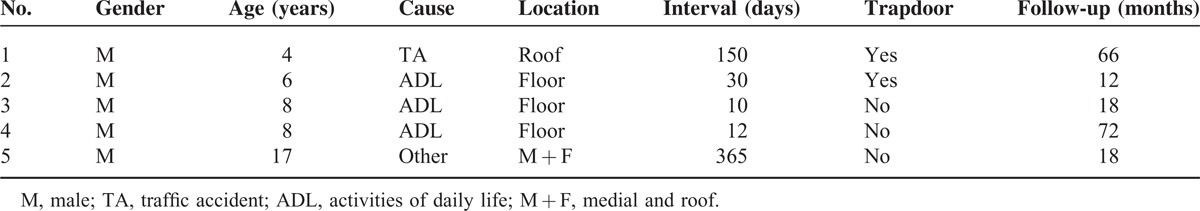
Characteristics of Patients Who Still Had Obvious Diplopia After Surgery

Five patients had postoperative diplopia of Grade 2 or Grade 3. It was noteworthy that 4/5 patients were very young, which suggested poor recovery in younger patients. Among them, 2 were wearing a prism to relieve diplopia, 1 was waiting for strabismus surgery, and the others refused surgical treatment and were closely followed-up. The percentage of patients showing full recovery was not significantly correlated with the age group (*P* = 0.11).

Among the 13 patients who had diplopia in either peripheral vision or forwards/downwards, a logistic regression analysis was performed to identify possible parameters related to residual diplopia (Table 5). Interval time was a parameter that was associated with postoperative diplopia (*P* = 0.02), but trapdoor fractures and patient age were parameters that were not correlated with diplopia after surgery.

**TABLE 5:**
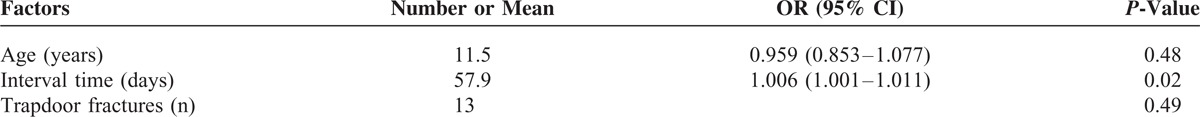
Logistic Regression Analysis of Possible Factors Related to Residual Diplopia

The approximate time for disappearance of diplopia after surgery was recorded during follow-up, with an average of 5.9 months, ranging from 3 days to 3 years. There was a significant difference in the remission time among the 3 age groups. Within these 3 groups, a significant difference was only found between the eldest group and 0- to 6-year-old patients (*P* < 0.01), and between the eldest group and 7- to 12-year-old patients (*P* = 0.02).

## DISCUSSION

An understanding of orbital blowout fractures in children is still evolving. However, early diagnosis and treatment are nonetheless important in preventing severe complications such as diplopia, restriction of ocular motility, and enophthalmos.^16^ Blowout fractures, which usually lead to entrapment of soft tissue and muscle due to the greater elasticity of the orbital bones, are considered a common type of orbital fracture in the young population.^5,6^

In the present study, traffic accidents, including both motor vehicle and bicycle accidents, were the most common cause of injury. This result was inconsistent with other reports, where sports, falls, motor vehicle accidents, or assaults were the most common causes.^17–19^ In China, especially in rural areas, proper safety equipment such as seatbelts, child seats, and helmets are not routinely used in motor or non-motor vehicles, which probably leads to a higher percentage of facial or orbital injuries in children. In addition, bicycle-related accidents are a major cause of injury. Zhang et al^20^ reported that 28.5% of pediatric maxillofacial injuries in Southern China were caused by bicycle-related accidents. Riding bicycles is more than a sport in China. It is also an important means of transportation, and numerous young people, especially in middle school or high school, ride bicycles between home and school, which may explain the different causes in pediatric orbital fractures.

Falls as a cause of orbital fractures declined with age, while sports injury as a cause of orbital fractures increased with age. These trends may be related to the enhancement of balance with increased involvement in sports activities. Assault as a major cause of orbital fractures only occurred in the eldest age group, which was probably the result of more interactions between people.

The structural characteristics of the pediatric orbit correlated with the orbital patterns. The orbital floor has been considered as the most frequent location of pediatric fractures.^9^ The most common location of fracture in this study was the orbital floor in the 0- to 6-year-old group and the 7- to 12-year-old group, while medial-floor fractures were the most common location in the 13- to 18-year-old group. Only the conclusion for the oldest age group was inconsistent with previous reports. However, age differences could explain these differences. The mean age of the group in the present study (15.6 years) was greater than in other reports by Hatton et al^18^ (12.5 years), Egbert et al^21^ (12.4 years), and Bansagi and Meyer^9^ (11.8 years). Children in the oldest age group reach an adult configuration with increased size of the midface, with pneumatization of the frontal sinus. Therefore, like adults, they were more likely to experience medial-floor fractures.

The number of patients who suffered roof-related fractures (roof only or roof with medial walls) decreased with increasing age. The relatively flat structure of the orbital roof in younger children will lead to more injury to the roof. Without arcing curves like adults, the orbital roof fractures more easily, because it cannot decompose the force from injury. The relatively high cranial:facial ratio (8:1) and the incomplete pneumatization of the frontal sinus may also contribute to this trend.

Diplopia is a common complication of blowout fractures. The evaluation of diplopia is difficult in the pediatric population, especially for children less than 5-years-old, who cannot clearly express themselves. However, the Hess test is a relatively easy and accurate way to evaluate diplopia in these patients. Furuta et al^22^ evaluated diplopia in 113 patients with orbital blowout fractures, including 8 patients from 0 to 9 years old and 36 patients from 10 to 19 years old, using the percentage of the Hess area ratio (HAR%), based on the Hess chart. HAR% is a numerical value and can be used to objectively evaluate diplopia and compare changes in patients. Thus, we suggest that the Grade 0 to Grade 3 classification of diplopia is a simple and direct method to classify the severity and change of diplopia, and its impact on livelihood.

Before surgery, an overall ophthalmic examination, including ocular vision, optic nerve function, diplopia, ocular motility, and forced duction testing, is strongly recommended in younger patients, especially less than 6 years of age, because children of this age lack the ability to fully express their symptoms, making diplopia difficult to diagnose. However, early treatment of orbital fractures is still recommended, as it has been in previous studies, especially with symptoms of nausea/vomiting, pain with ocular motility, or signs of oculocardiac reflex.^21,23^

In the present study, the characteristics of clinical presentation in pediatric orbital blowout fractures varied among the 3 age groups, which should assist clinicians when treating pediatric patients of different ages. In younger patients, enophthalmos occurred less frequently than adults, probably because the greater elasticity of the orbital bone could result in “trapdoor fractures,” with the volume of orbit not greatly enlarged. Diplopia and evidence of entrapment of muscle or soft tissue were the main clinical problems.

Based upon the present study, for younger patients, the poor outcomes and longer recovery times of diplopia compared with older patients should be stressed. Early intervention is always recommended, and functional training of the eye muscles should be performed soon after surgery. If necessary, correct training methods should be taught to parents and/or medical staff.

The characteristics of our older patients are similar to those of adults. Enophthalmos, together with diplopia and ocular motility restriction, are more common. Thus, both functional (diplopia) and aesthetic (enophthalmos) problems should be addressed. Precise reconstruction of the orbital volume is very important when dealing with enophthalmos. Advanced surgical technology such as the navigation system is an ideal method to reconstruct the bony orbit and optimize treatment outcomes.

Persistence of diplopia after surgical repair is always a problem. A number of potential factors related to residual diplopia in the adult population have been previously reported, including fracture volume,^24^ ocular muscle swelling,^25^ and preoperative diplopia.^26^ Tahiri et al^26^ reported that 26.4% of adult patients presented with diplopia 6 months after surgical repair. In our study, 13/83 (15.7%) pediatric patients had residual diplopia after surgery, including 5 patients with diplopia, which significantly affected their daily life. The important parameter associated with residual diplopia was the interval time between injury and surgical repair, which again emphasized the importance of early intervention in the surgical repair of orbital blowout fractures.

The results of this study should be considered with several limitations. Although this study was comprised of a relatively large series of pediatric patients, and tried to subcategorize them by age groups, the number of patients in each group was small. In addition, the pediatric patients only included those who needed surgical treatment. Children with orbital fracture but without surgical indications were not studied. Lastly, our ophthalmology department mainly enrolled patients from the eastern areas of China, so there could possibly be some geographical bias in our results.

In summary, characteristics of orbital blowout fractures in different pediatric age groups were described in our study. The common causes of injury and fracture locations in each age group were not distinct, but were probably age-related, and structural modifications in growth and changes in the habits of daily life in the different age groups could have possibly explained the differences. Regarding the clinical outcomes of children, our study was consistent with other studies that reported that children recover more slowly.^27,28^ For patients from the 0- to 6-years-old group, the outcomes of diplopia were the poorest, and the time for disappearance of diplopia was the longest in the pediatric population.
